# The differences on efficacy of oxaliplatin in locally advanced colon cancer between mucinous and nonmucinous adenocarcinoma

**DOI:** 10.1002/cam4.1333

**Published:** 2018-01-29

**Authors:** Dehao Yu, Peng Gao, Yongxi Song, Yuchong Yang, Xiaowan Chen, Yu Sun, Ailin Li, Zhenning Wang

**Affiliations:** ^1^ Department of Surgical Oncology and General Surgery The First Hospital of China Medical University 155 North Nanjing Street Heping District Shenyang City 110001 China; ^2^ Department of Radiation Oncology The First Hospital of China Medical University 155 North Nanjing Street Heping District Shenyang City 110001 China

**Keywords:** Chemotherapy, colon neoplasms, mucinous adenocarcinoma, nonmucinous adenocarcinoma, SEER program

## Abstract

Until now, it remains unclear how to best use the histological subtype in clinical practice. This study aimed to compare differences in the efficacy of postoperative chemotherapy among different histological subtypes of colon adenocarcinomas. Using the Surveillance, Epidemiology, and End Results‐Medicare database, 51,200 patients with stage II or III primary colon carcinomas who underwent resection for curative intent between 1992 and 2008 were included. The survival benefit was evaluated using a Cox proportional hazards model, interaction analyses, and propensity score‐matched techniques. There was no significant difference in survival for low‐risk stage II mucinous adenocarcinoma (MA) or nonmucinous adenocarcinoma (NMA) between 5‐FU and oxaliplatin‐treated groups (*P *=* *0.387 for MA,* P *=* *0.629 for NMA). Patients with high‐risk stage II NMA who received the oxaliplatin chemotherapy regimen had significantly improved cancer‐specific survival (CSS) compared with the 5‐FU group (*P *=* *0.004), while those with MA saw no improvement (*P *=* *0.690). For stage III tumors, patients with NMA who received the oxaliplatin chemotherapy regimen had significantly improved CSS compared with the 5‐FU group (*P *<* *0.001), while those with MA saw no improvement (*P *=* *0.300). There were significant interactions between chemotherapy regimen and histological subtype. For patients with resected colon cancer who received 5‐FU‐based postoperative chemotherapy, oxaliplatin chemotherapy prolongs CSS for stage III and high‐risk stage II NMA. Conversely, there was no similar improvement with addition of oxaliplatin for patients with stage III or stage II MA.

## Introduction

The use of histological subtype as a classification system for colorectal cancer was introduced by the World Health Organization in 1979. Carcinomas are categorized as traditional adenocarcinomas, mucinous adenocarcinomas (MA), signet‐ring cell carcinomas (SC), and other, more infrequent, types 1, 2. MA is a histological subtype of colon cancer in which the neoplastic cells secrete extensive extracellular mucins that form more than 50% of the tumor volume 3. SC tumors are comprised of more than 50% signet‐ring cells in which the nucleus is pushed to the periphery by intracytoplasmic mucins of colon cancer 4. This classification of histological subtype is routinely carried out during the postoperative pathological examination of colon cancer. However, how to this histological subtyping should best be used to aid in the clinical practice remains unclear.

In clinical practice, decision making regarding whether give or which regimen give adjuvant therapy to patients with stage II tumors remains controversial 5, 6. For patients with stage III disease, although the preferred treatment options are FOLFOX or CapeOx, the side effects of oxaliplatin are indisputable. It has been reported that oxaliplatin might not be applicable for all patients, specifically the elderly population 7, 8. Thus, it is important to find prognostic and predictive features to help assist with selecting appropriate and beneficial adjuvant therapy for patients considered. Histological subtype is not considered in the decision making for colon cancer adjuvant therapy in either the National Comprehensive Cancer Network (NCCN) 9 or the European Society for Medical Oncology (ESMO) 10. In addition, no research has proposed that histological subtype could have an influence on chemotherapeutic effects in colorectal cancer patients. As for other types of cancer, Sugawa et al. 11 found a difference in chemotherapy effects between different histological subtypes in cervical cancer, and Itaya et al. 12 found histology‐dependent differences of chemosensitivity in nonsmall cell lung cancer.

The aim of this study was to compare the efficacy of postoperative chemotherapy among different histological subtypes of colon cancer. We then tried to find the most suitable postoperative chemotherapy regimens for both major histological subtypes of colonic adenocarcinoma.

## Materials and Methods

### Data source

This study was a retrospective analysis of data collected from the Surveillance, Epidemiology, and End Results (SEER)–Medicare linked database. This study was conducted in accordance with a SEER‐Medicare data use agreement, and a study protocol approval was also granted by the First Hospital of China Medical University Institutional Review Board.

SEER data contain information on patient demographics, tumor and disease characteristics, course of treatment, use of cancer‐directed operative and medical therapy, survival, and cause of death for individuals diagnosed with cancer. It is a population‐based cancer registry covering approximately 28% of the US population across several disparate geographic regions 13. Medicare is the primary health insurer for approximately 97% of the US population aged ≥65 years 14. The unmentioned details of the database appeared elsewhere 15.

### Patient selection

All Medicare‐registered patients diagnosed with incident malignant primary colon cancer (SEER cancer site codes: 18.0, 18.2–18.9) between 1992 and 2008 in a SEER area were considered for study inclusion. The study contained two histological types defined by WHO International Classification of Diseases for Oncology, 3rd edition (ICD‐O‐3), codes: MA (8480) and nonmucinous adenocarcinomas (NMA) (8010, 8020–8022, 8140–8141, 8144–8145, 8210–8211, 8220–8221, 8230–8231, 8260–8263).

Patients were selected who underwent primary tumor resection with likely curative intent within 180 days of diagnosis. The No‐chemo group was designated as no claim of postoperative chemotherapy within 9 months after operation. The 5‐FU group consisted of patients who only received 5‐FU/capecitabine chemotherapy within 9 months of surgery. The oxaliplatin group comprised patients with any record of oxaliplatin plus 5‐FU/capecitabine within 9 months of surgery.

Patients were eliminated from the study population if they (1) received any preoperative adjuvant treatment; (2) received postoperative radiotherapy; (3) had prior noncolon cancer; (4) had incomplete histological subtype or pathological stage entries; (5) died within 30 days after tumor resection; (6) had stage I or stage IV tumors; (7) histological subtype was signet‐ring cell carcinoma, as this population represented too small a sample size (0.9%).

### Variables

Subjects were categorized by age at diagnosis, year of diagnosis, gender, race, marital status, residence (rural or urban), median household income, level of education (percentage of people aged >25 years with <12 years of education), and the type of hospital in which they received care (teaching or nonteaching). To control for the effects of comorbidities, analyses were adjusted by the Centers for Medicare and Medicaid Services Hierarchical Condition Category (HCC) based on Medicare outpatient and inpatient claims for miscellaneous comorbidities within the 12 months before colon cancer diagnosis. The HCC risk score summarizes the healthcare problems and forecasts the future healthcare cost of a population compared with the average Medicare beneficiary 16.

Postoperative pathological stage was designated via the seventh edition of the Union for International Cancer Control (UICC) tumor‐node‐metastasis (TNM) staging system 17. Other covariates included histological grade, histological subtype, intestinal obstruction, intestinal perforation, and the number of lymph nodes examined.

### Statistical analysis

The chi‐square test was used to compare demographics and tumor characteristics between the different groups. In the univariate survival analysis, cancer‐specific survival (CSS) was analyzed by the Kaplan–Meier method. Comparison of survival curves was carried out using the log‐rank test. Because treatment choice estimates are likely confounded by factors related to treatment selection, a propensity score (PS)‐matched analysis was performed to compare the effect of treatment on survival among patients of similar risk profiles as assessed by measured known confounders 18, 19. Propensity score matching is a statistical procedure for reducing this bias by assembling a sample in which confounding factors are balanced between treatment groups. Univariate logistic regression was used to find factors related to treatment selection (*P *<* *0.05). Multivariate logistic regression was used to estimate the propensity scores in each group (Table 1). The propensity score‐matched sample would then be constructed using “psmatch2” software package in STATA 14.0. A Cox proportional hazards model was also used in the adjusted analysis. The covariates included all variables that were identified to be significantly related to survival in the univariate analysis.

**Table 1:** Main effect variables in propensity score models

NMA Patients in low‐risk stage II	
Variables that significantly related to the patients’ probability of receiving 5‐FU compared with No‐chemo	Gender, age at diagnosis, year at diagnosis, HCC risk score, race, marital status
MA Patients in low‐risk stage II	
Variables that significantly related to the patients’ probability of receiving 5‐FU compared with No‐chemo	Gender, age at diagnosis, year at diagnosis
NMA Patients in high‐risk stage II	
Variables that significantly related to the patients’ probability of receiving 5‐FU compared with No‐chemo	Gender, age at diagnosis, year at diagnosis, histological grade, pT category, intestinal obstruction, HCC risk score, number of examined lymph node, level of education, marital status, residence location
Variables that significantly related to the patients’ probability of receiving 5‐FU plus oxaliplatin compared with 5‐FU alone	Age at diagnosis, year at diagnosis, pT category, number of examined lymph node, median income, marital status
MA Patients in high‐risk stage II	
Variables that significantly related to the patients’ probability of receiving 5‐FU compared with No‐chemo	Gender, age at diagnosis, year at diagnosis, pT category, intestinal obstruction, HCC risk score, marital status, profit hospital
Variables that significantly related to the patients’ probability of receiving 5‐FU plus oxaliplatin compared with 5‐FU alone	Year at diagnosis, pT category, number of examined lymph node, profit hospital
NMA Patients in stage III	
Variables that significantly related to the patients’ probability of receiving 5‐FU compared with No‐chemo	Gender, age at diagnosis, year at diagnosis, pT category, pN category, intestinal obstruction, HCC risk score, level of education, median income, race, marital status, residence location
Variables that significantly related to the patients’ probability of receiving 5‐FU plus oxaliplatin compared with 5‐FU alone	Age at diagnosis, year at diagnosis, pN category, number of examined lymph node, level of education, median income, marital status
MA Patients in stage III	
Variables that significantly related to the patients’ probability of receiving 5‐FU compared with No‐chemo	Gender, age at diagnosis, year at diagnosis, pT category, intestinal obstruction, HCC risk score, marital status
Variables that significantly related to the patients’ probability of receiving 5‐FU plus oxaliplatin compared with 5‐FU alone	Gender, age at diagnosis, year at diagnosis, pN category, number of examined lymph node, median income, marital status

MA, mucinous adenocarcinoma; NMA, nonmucinous adenocarcinoma; HCC, hierarchical condition categories; 5‐FU, 5‐fluorouracil.

All statistical analyses and graphics were performed using SAS 9.4 (SAS Institute, Cary, NC), STATA 14.0 software (STATA, College Station, TX), and PASW Statistics 20.0 software (SPSS, Inc., Somers, NY). For all analyses, a *P* value < 0.05 was considered statistically significant.

## Results

### Patient characteristics

Selected 51,200 individuals were stratified into two analysis groups: NMA (*n* = 43,998) and MA (*n* = 7202). Demographic characteristics of patients are depicted in Table 2. Compared with NMA, MA was more common in women (*P *<* *0.001), individuals aged >80 years (*P *=* *0.018), year at diagnosis before 2004 (*P *<* *0.001), well histological grade (*P *<* *0.001), T1–T3 category (*P *<* *0.001), N2 category (*P *<* *0.001), nonintestinal obstruction (*P *<* *0.001), number of examined lymph nodes ≥12 (*P *<* *0.001), white race (*P *<* *0.001), widowed (*P *=* *0.006). Stage II patients were further divided into low‐risk stage II and high‐risk stage II groups. We designated the cohort of patients with high‐risk stage II using features of poor prognosis referred to in the NCCN 9, including T4 tumors, poorly differentiated histology, bowel obstruction, bowel perforation, and inadequate sampled nodes (<12 lymph nodes). The number of patients and the results of each analysis and treatment chemotherapy effect analysis are summarized in Table 3.

**Table 2:** Clinicopathologic features of patients with different histological subtype

	NMA	MA	*P*
	*n* (%)	*n* (%)	
Gender			<0.001
Male	18,479 (42.0%)	2665 (37.0%)	
Female	25,519 (58.0%)	4537 (63.0%)	
Age at diagnosis, years			0.018
<70	6712 (15.3%)	1027 (14.3%)	
70–74	8840 (20.1%)	1411 (19.6%)	
75–79	10,066 (22.9%)	1610 (22.4%)	
80–84	9205 (20.9%)	1563 (21.7%)	
>84	9175 (20.9%)	1591 (22.1%)	
Year at diagnosis			0.001
1992–1996	8818 (20.0%)	1472 (20.4%)	
1997–2000	8393 (19.1%)	1421 (19.7%)	
2001–2004	14,410 (32.8%)	2452 (34.0%)	
2005–2008	12,377 (28.1%)	1857 (25.8%)	
Histological grade			<0.001
Well	2695 (6.1%)	658 (9.1%)	
Moderate	30,354 (69.0%)	4470 (62.1%)	
Poor	9707 (22.1%)	1475 (20.5%)	
Undifferentiated	503 (1.1%)	77 (1.1%)	
Unknown	739 (1.7%)	522 (7.2%)	
Postoperative chemotherapy			0.396
No	28,104 (63.9%)	4563 (63.4%)	
Yes	15,894 (36.1%)	2639 (36.6%)	
pT category			<0.001
T1	612 (1.4%)	57 (0.8%)	
T2	1504 (3.4%)	197 (2.7%)	
T3	35,209 (80.0%)	5661 (78.6%)	
T4a	4134 (9.4%)	787 (10.9%)	
T4b	2539 (5.8%)	500 (6.9%)	
pN category			<0.001
N0	24,869 (56.5%)	4105 (57.0%)	
N1a	6852 (15.6%)	1016 (14.1%)	
N1b	6422 (14.6%)	992 (13.8%)	
N2a	3652 (8.3%)	604 (8.4%)	
N2b	2203 (5.0%)	485 (6.7%)	
Intestinal obstruction			<0.001
No	34,677 (78.8%)	5910 (82.1%)	
Yes	9321 (21.2%)	1292 (17.9%)	
Intestinal perforation			0.463
No	43,401 (98.6%)	7112 (98.8%)	
Yes	597 (1.4%)	90 (1.2%)	
HCC risk score			0.001
1st quartile	11,575 (26.3%)	1974 (27.4%)	
2nd quartile	10,846 (24.7%)	1707 (23.7%)	
3rd quartile	10,892 (24.8%)	1671 (23.2%)	
4th quartile	10,685 (24.3%)	1850 (25.7%)	
Number of examined lymph node			<0.001
<12	20,747 (47.2%)	3164 (43.9%)	
≥12	23,251 (52.8%)	4038 (56.1%)	
Level of education			0.712
1st quartile	11,129 (25.3%)	1845 (25.6%)	
2nd quartile	11,088 (25.2%)	1828 (25.4%)	
3rd quartile	10,974 (24.9%)	1772 (24.6%)	
4th quartile	8899 (20.2%)	1426 (19.8%)	
Unknown	1908 (4.3%)	331 (4.6%)	
Median income			0.061
1st quartile	10,858 (24.7%)	1815 (25.2%)	
2nd quartile	11,072 (25.2%)	1698 (23.6%)	
3rd quartile	11,063 (25.1%)	1828 (25.4%)	
4th quartile	9097 (20.7%)	1530 (21.2%)	
Unknown	1908 (4.3%)	331 (4.6%)	
Race			<0.001
White	37,285 (84.7%)	6279 (87.2%)	
Black	3859 (8.8%)	560 (7.8%)	
Asian	1303 (3.0%)	149 (2.1%)	
Others	1551 (3.5%)	214 (3.0%)	
Marital status			0.006
Single	4042 (9.2%)	636 (8.8%)	
Married	20,997 (47.7%)	3341 (46.4%)	
Widowed	17,487 (39.7%)	3011 (41.8%)	
Others	1472 (3.3%)	214 (3.0%)	
Residence location^a^			0.127
Big Metro	23,585 (53.6%)	3938 (54.7%)	
Metro or Urban	15,298 (34.8%)	2416 (33.5%)	
Less Urban or Rural	5113 (11.6%)	848 (11.8%)	
Profit hospital^a^			0.006
Nonprofit hospital	21,362 (49.4%)	3620 (51.1%)	
For‐profit hospital	15,938 (36.9%)	2586 (36.5%)	
Public hospital	5924 (13.7%)	885 (12.5%)	
Number of beds^a^			0.076
1st quartile	10,653 (24.6%)	1772 (25.0%)	
2nd quartile	10,795 (25.0%)	1823 (25.7%)	
3rd quartile	10,819 (25.0%)	1800 (25.4%)	
4th quartile	10,957 (25.3%)	1696 (23.9%)	
Teaching hospital^a^			0.941
Yes	22,604 (52.7%)	3699 (52.6%)	
No	20,304 (47.3%)	3329 (47.4%)	

HCC, hierarchical condition categories; NMA, nonmucinous adenocarcinomas; MA, mucinous adenocarcinomas.

a Variables have missing data.

**Table 3:** Results of patients subjected to different chemotherapy regimens

	Number of patients			HR	95% CI	*P*
	No‐chemo	5‐FU	Oxaliplatin			
Low‐risk stage II						
No‐PSM‐NMA (No‐ chemo vs. 5‐FU)	5958	961	–	0.735	0.604–0.893	0.002
No‐PSM‐NMA (5‐FU vs. oxaliplatin)	–	961	94	0.462	0.146–1.465	0.179
No‐PSM‐MA (No‐ chemo vs. 5‐FU)	1025	178	–	0.934	0.582–1.496	0.775
No‐PSM‐MA (5‐FU vs. oxaliplatin)	–	178	13	0.045	0.001–843.46	0.346
PSM‐NMA (No‐chemo vs. 5‐FU)	961	961	–	0.939	0.726–1.214	0.629
PSM‐MA (No‐chemo vs. 5‐FU)	176	176	–	1.399	0.690–2.598	0.387
High‐risk stage II						
No‐PSM‐NMA (No‐chemo vs. 5‐FU)	13,951	2664	–	0.826	0.758–0.901	<0.001
No‐PSM‐NMA (5‐FU vs. oxaliplatin)	–	2664	260	0.529	0.348–0.804	0.002
No‐PSM‐MA (No‐chemo vs. 5‐FU)	2028	443	–	0.749	0.598–0.938	0.011
No‐PSM‐MA (5‐FU vs. oxaliplatin)	–	443	37	0.792	0.289–2.172	0.649
PSM‐NMA (No‐chemo vs. 5‐FU)	2662	2662	–	1.003	0.894–1.125	0.961
PSM‐NMA (5‐FU vs. oxaliplatin)	–	260	260	0.529	0.348–0.804	0.004
PSM‐MA (No‐chemo vs. 5‐FU)	436	436	–	1.049	0.778–1.415	0.754
PSM‐MA (5‐FU vs. oxaliplatin)	–	29	29	0.792	0.289–2.172	0.690
Stage III						
No‐PSM‐NMA (No‐chemo vs. 5‐FU)	7843	8188	–	0.551	0.525–0.578	<0.001
No‐PSM‐NMA (5‐FU vs. oxaliplatin)	–	8188	1826	0.583	0.522–0.625	<0.001
No‐PSM‐MA (No‐chemo vs. 5‐FU)	1287	1360	–	0.566	0.503–0.637	<0.001
No‐PSM‐MA (5‐FU vs. oxaliplatin)	–	1360	258	0.74	0.569–0.962	0.023
PSM‐NMA (No‐chemo vs. 5‐FU)	7841	7841	–	0.554	0.527–0.581	<0.001
PSM‐NMA (5‐FU vs. oxaliplatin)	–	1819	1819	0.621	0.543–0.710	<0.001
PSM‐MA (No‐chemo vs. 5‐FU)	1287	1287	–	0.567	0.502–0.639	<0.001
PSM‐MA (5‐FU vs. oxaliplatin)	–	252	252	0.837	0.598–1.173	0.300

PSM, propensity score matched; MA, mucinous adenocarcinoma; NMA, nonmucinous adenocarcinoma; HR, hazard ratio; CI, confidential intervals; 5‐FU, 5‐fluorouracil; No‐chemo, without postoperative chemotherapy.

### CSS in low‐risk stage II adenocarcinoma

**Figure 1:**
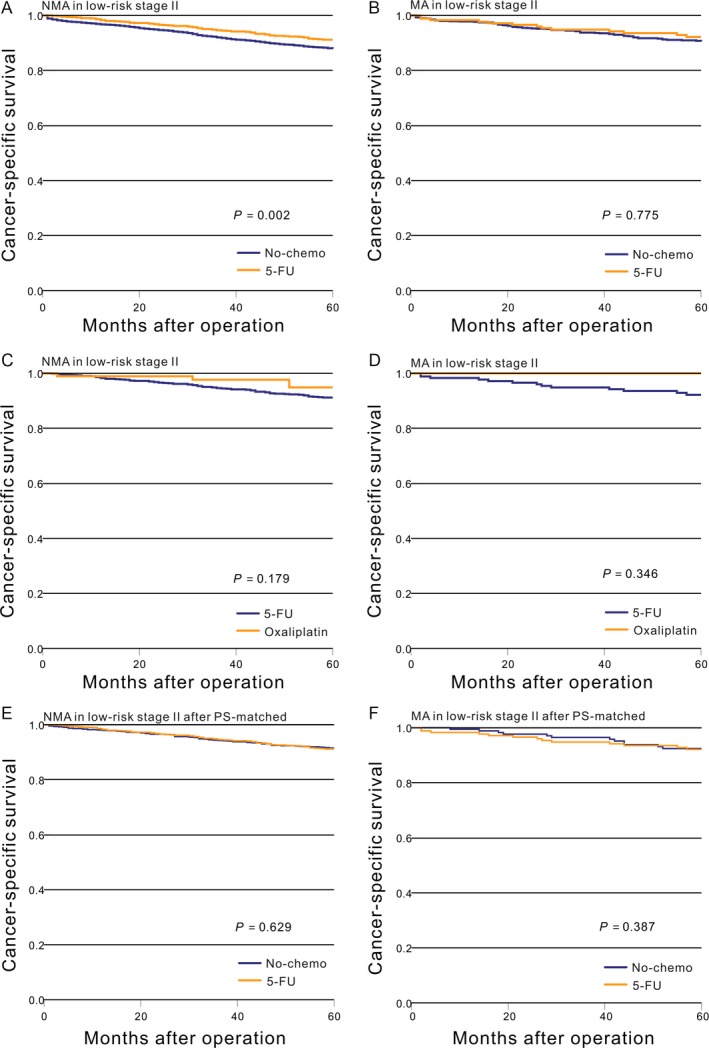
Kaplan–Meier comparison of cancer‐specific survival among patients who received different postoperative treatment stratified by histological subtype. (A) NMA in low‐risk stage II (No‐chemo vs. 5‐FU); (B) MA in low‐risk stage II (No‐chemo vs. 5‐FU); (C) NMA in low‐risk stage II (5‐FU vs. oxaliplatin); (D) MA in low‐risk stage II (5‐FU vs. oxaliplatin); (E) NMA in low‐risk stage II after PS‐matched (No‐chemo vs. 5‐FU); (F) MA in low‐risk stage II after PS‐matched (No‐chemo vs. 5‐FU).

There was a significant difference in survival for NMA patients with low‐risk stage II cancer between the no‐chemo and 5‐FU groups (*P *=* *0.002, Fig. 1A), while those with MA saw no difference (*P *=* *0.775, Fig. 1B). There was no significant difference in NMA and MA patients with low‐risk stage II cancer between the 5‐FU and oxaliplatin groups (Fig. 1C and D).

A PS‐matched cohort was generated using related variables which may interfere with the chemotherapy decision (Table 1). The aforementioned general results were recalculated in the PS‐match cohorts. There was no significant difference in survival for patients with low‐risk stage II NMA between the no‐chemo and 5‐FU groups (*P *=* *0.629, Fig. 1E), while those with MA again saw no difference (*P *=* *0.387, Fig. 1F). Another PS‐matched cohort was generated using related variables which may interfere with the choice of chemotherapy regimen. However, its sample size is too small to recalculate aforementioned results.

### CSS in high‐risk stage II adenocarcinoma

**Figure 2:**
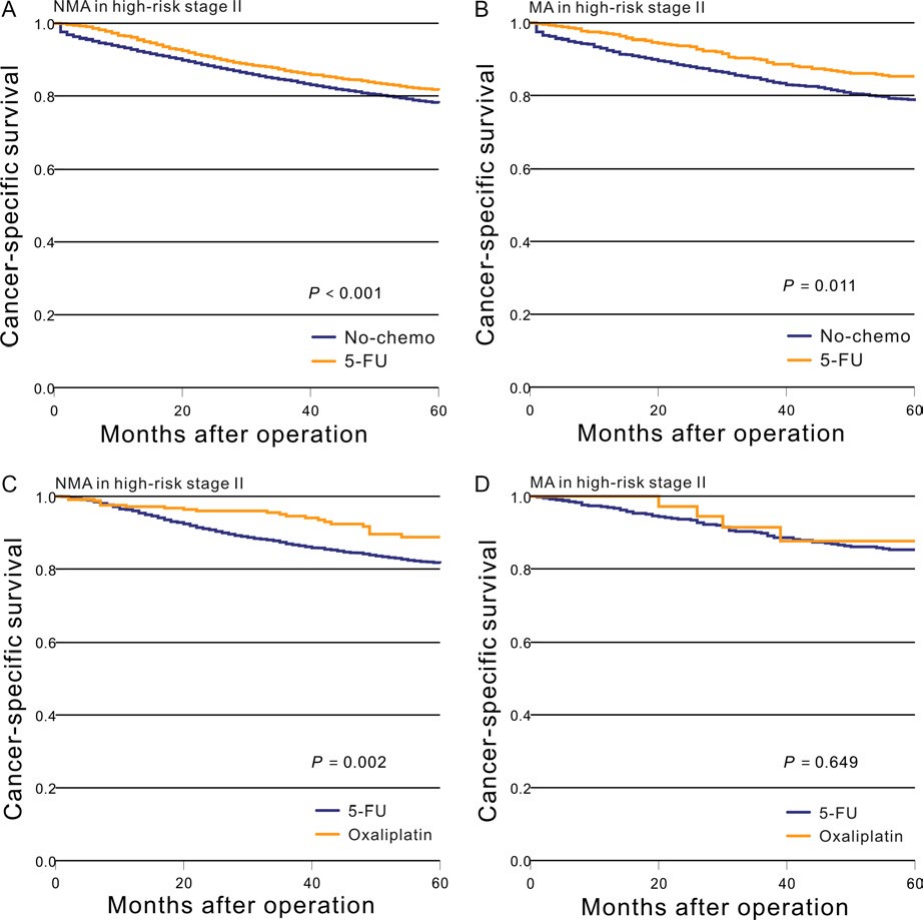
Kaplan–Meier comparison of cancer‐specific survival among patients who received different postoperative treatment stratified by histological subtype. (A) NMA in high‐risk stage II (No‐chemo vs. 5‐FU); (B) MA in high‐risk stage II (No‐chemo vs. 5‐FU); (C) NMA in high‐risk stage II (5‐FU vs. oxaliplatin); (D) MA in high‐risk stage II (5‐FU vs. oxaliplatin).

There was a significant difference in survival for patients with high‐risk stage II NMA between the no‐chemo and 5‐FU groups (*P *<* *0.001, Fig. 2A), while those with MA again saw a difference (*P *=* *0.011, Fig. 2B). Patients with NMA who received the oxaliplatin chemotherapy regimen had significantly improved CSS (*P *=* *0.002, Fig. 2C) compared with the 5‐FU group, while those with MA saw no improvement (*P *=* *0.649, Fig. 2D).

**Figure 3:**
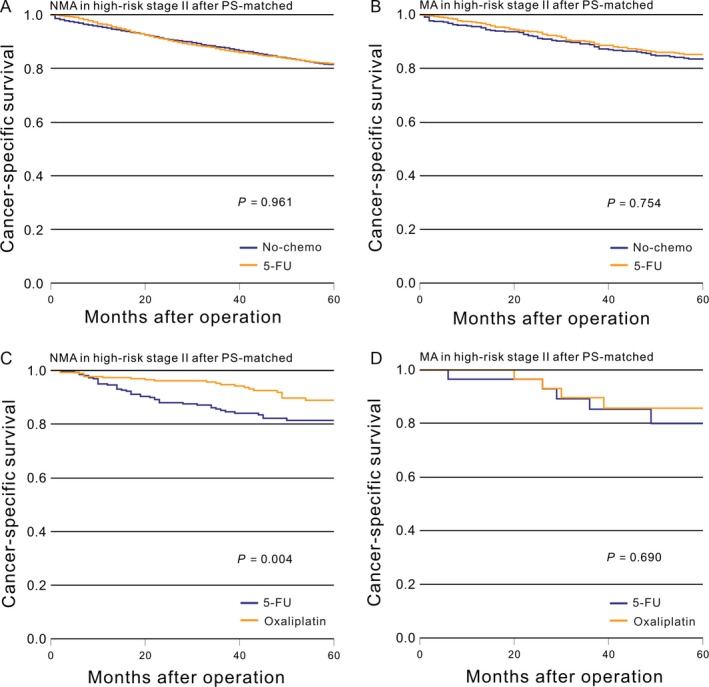
After PS‐matched, Kaplan–Meier comparison of cancer‐specific survival among patients who received different postoperative treatment stratified by histological subtype. (A) NMA in high‐risk stage II (No‐chemo vs. 5‐FU); (B) MA in high‐risk stage II (No‐chemo vs. 5‐FU); (C) NMA in high‐risk stage II (5‐FU vs. oxaliplatin); (D) MA in high‐risk stage II (5‐FU vs. oxaliplatin).

Then, we used the PS‐match cohorts to recalculate the aforementioned general results. There was no significant difference in survival for patients with high‐risk stage II NMA between the no‐chemo and 5‐FU groups (*P *=* *0.961, Fig. 3A), while those with MA again saw no difference (*P *=* *0.754, Fig. 3B). Patients with NMA who received the oxaliplatin chemotherapy regimen had significantly improved CSS (*P *=* *0.004, Fig. 3C) compared with the 5‐FU group, while those with MA saw no improvement (*P *=* *0.690, Fig. 3D). This result was also verified by a Cox proportional hazards model (Table 4 and 5).

**Table 4:** Cox proportional hazards model for patients in high‐risk stage II stratified by histological subtype

	HR	95% CI	*P*
NMA			
Chemotherapy regimen			
5‐FU	1		
Oxaliplatin	0.867	0.564–0.366	0.009
Intestinal perforation			
No	1		
Yes	2.165	1.449‐0.970	0.070
Intestinal obstruction			
No	1		
Yes	1.619	1.356–1.135	0.001
Number of examined lymph node			
<12	1		
≥12	0.817	0.681–0.568	<0.001
HCC risk score			
1st quartile	1		
2nd quartile	1.584	1.227–0.950	0.118
3rd quartile	1.679	1.303–1.010	0.041
4th quartile	1.692	1.289–0.982	0.068
Race			
White	1		
Black	2.025	1.504–1.116	0.007
Asian	0.863	0.472–0.258	0.015
Others	1.167	0.715–0.438	0.179
pT category			
T3	1		
T4a	1.739	1.383–1.100	0.005
T4b	3.419	2.763–2.233	<0.001
Age at diagnosis, years			
<70	1		
70–74	1.653	1.311–1.039	0.022
75–79	1.947	1.543–1.222	<0.001
80–84	2.533	1.917–1.450	<0.001
>84	5.814	3.870–2.577	<0.001
Marital status			
Single	1		
Married	1.028	0.780–0.591	0.078
Widowed	1.343	1.005–0.752	0.973
Others	1.460	0.851–0.496	0.557
Level of education			
1st quartile	1		
2nd quartile	1.259	1.002–0.798	0.984
3rd quartile	1.134	0.903–0.719	0.380
4th quartile	1.266	0.995–0.782	0.969
Gender			
Male	1		
Female	1.133	0.938–0.777	0.507
MA			
Chemotherapy regimen			
5‐FU	1		
Oxaliplatin	0.622	0.224–1.732	0.364
Intestinal perforation			
No	1		
Yes	1.827	0.712–4.683	0.210
Intestinal obstruction			
No	1		
Yes	1.050	0.634–1.738	0.851
pT category			
T3	1		
T4a	1.543	0.910–2.616	0.108
T4b	3.666	2.244–5.990	<0.001
Age at diagnosis, years			
<70	1		
70–74	0.870	0.500–1.513	0.622
75–79	0.985	0.550–1.762	0.959
80–84	1.816	0.912–3.615	0.090
>84	2.731	1.204–6.199	0.016
Marital status			
Single	1		
Married	0.563	0.292–1.088	0.087
Widowed	0.517	0.253–1.060	0.072
Others	0.227	0.028–1.837	0.165

MA, mucinous adenocarcinoma; NMA, nonmucinous adenocarcinoma; HCC, hierarchical condition categories; HR, hazard ratio; CI, confidential intervals; 5‐FU, 5‐fluorouracil.

**Table 5:** Univariate prognostic analysis stratified by histological subtype

	HR	95% CI	*P*
NMA in stage III			
Age at diagnosis, years			
<70	1		
70–74	1.070	0.991–1.155	0.082
75–79	1.217	1.130–1.311	<0.001
80–84	1.468	1.361–1.583	<0.001
>84	2.021	1.875–2.179	<0.001
Year at diagnosis			
1992–1996	1		
1997–2000	1.000	0.934–1.070	0.989
2001–2004	0.925	0.870–0.984	0.014
2005–2008	0.850	0.794–0.910	<0.001
HCC risk score			
1st quartile	1		
2nd quartile	0.838	0.787–0.891	<0.001
3rd quartile	0.905	0.850–0.964	0.002
4th quartile	1.117	1.050–1.189	<0.001
Number of examined lymph node			
<12	1		
≥12	0.890	0.851–0.930	<0.001
pT category			
T1	1		
T2	1.240	0.994–1.547	0.057
T3	2.804	2.311–3.401	<0.001
T4a	4.124	3.375–5.039	<0.001
T4b	7.451	6.087–9.120	<0.001
Intestinal perforation			
No	1		
Yes	2.259	1.928–2.648	<0.001
Intestinal obstruction			
No	1		
Yes	1.549	1.473–1.629	<0.001
Marital status			
Single	1		
Married	0.793	0.733–0.858	<0.001
Widowed	1.007	0.930–1.091	0.864
Others	0.926	0.802–1.070	0.299
Chemotherapy regimen			
5‐FU	1		
Oxaliplatin	0.583	0.522–0.652	<0.001
Histological grade			
Well	1		
Moderate	1.264	1.123–1.422	<0.001
Poor	1.801	1.595–2.034	<0.001
Undifferentiated	1.902	1.550–2.334	<0.001
Unknown	1.223	0.993–1.507	0.059
Median income			
1st quartile	1		
2nd quartile	0.938	0.881–0.997	0.041
3rd quartile	0.905	0.850–0.963	0.002
4th quartile	0.858	0.803–0.918	<0.001
Unknown	1.027	0.919–1.148	0.639
pN category			
N1a	1		
N1b	1.417	1.337–1.503	<0.001
N2a	2.000	1.877–2.131	<0.001
N2b	3.272	3.056–3.503	<0.001
Race			
White	1		
Black	1.066	0.988–1.149	0.098
Asian	0.750	0.653–0.86	<0.001
Others	0.945	0.843–1.06	0.338
Level of education			
1st quartile	1		
2nd quartile	1.091	1.024–1.163	0.007
3rd quartile	1.140	1.069–1.215	<0.001
4th quartile	1.195	1.118–1.278	<0.001
Unknown	1.219	1.090–1.364	0.001
Gender			
Male	1		
Female	1.069	1.022–1.119	0.004
MA in stage III			
Age at diagnosis, years			
<70	1		
70–74	1.037	0.857–1.256	0.706
75–79	1.216	1.011–1.464	0.038
80–84	1.405	1.164–1.696	<0.001
>84	1.692	1.401–2.045	<0.001
Year at diagnosis			
1992–1996	1		
1997–2000	1.040	0.881–1.227	0.643
2001–2004	1.032	0.889–1.198	0.677
2005–2008	0.826	0.694–0.983	0.032
HCC risk score			
1st quartile	1		
2nd quartile	0.847	0.724–0.991	0.038
3rd quartile	0.986	0.844–1.151	0.854
4th quartile	1.203	1.037–1.396	0.015
Number of examined lymph node			
<12	1		
≥12	0.792	0.710–0.884	<0.001
pT category			
T1	1		
T2	0.841	0.445–1.590	0.595
T3	2.214	1.280–3.828	0.004
T4a	3.590	2.048–6.295	<0.001
T4b	5.753	3.270–10.122	<0.001
Intestinal obstruction			
No	1		
Yes	1.634	1.436–1.859	<0.001
Marital status			
Single	1		
Married	0.776	0.636–0.948	0.013
Widowed	0.941	0.770–1.151	0.555
Others	1.089	0.780–1.520	0.618
pN category			
N1a	1		
N1b	1.208	1.040–1.402	0.013
N2a	1.905	1.628–2.228	<0.001
N2b	2.752	2.344–3.231	<0.001
Intestinal perforation			
No	1		
Yes	1.915	1.151–3.188	0.012
Chemotherapy regimen			
5‐FU	1		
Oxaliplatin	0.740	0.569–0.962	0.025
Histological grade			
Well	1		
Moderate	0.955	0.763–1.197	0.692
Poor	1.332	1.053–1.685	0.017
Undifferentiated	1.701	1.073–2.697	0.024
Unknown	1.129	0.847–1.503	0.407
NMA in high‐risk stage II			
Intestinal perforation			
No	1		
Yes	2.666	2.249–3.161	<0.001
Chemotherapy regimen			
5‐FU	1		
Oxaliplatin	0.529	0.348–0.804	0.003
Intestinal obstruction			
No	1		
Yes	1.510	1.413–1.613	<0.001
Number of examined lymph node			
<12	1		
≥12	0.921	0.859–0.987	0.020
HCC risk score			
1st quartile	1		
2nd quartile	0.925	0.847–1.011	0.087
3rd quartile	1.045	0.958–1.141	0.321
4th quartile	1.286	1.178–1.404	<0.001
Race			
White	1		
Black	1.424	1.284–1.580	<0.001
Asian	0.698	0.560–0.870	0.001
Others	1.033	0.863–1.235	0.726
pT category			
T3	1		
T4a	1.357	1.238–1.489	<0.001
T4b	2.589	2.348–2.855	<0.001
Age at diagnosis, years			
<70	1		
70–74	0.969	0.858–1.094	0.612
75–79	1.210	1.079–1.358	0.001
80–84	1.416	1.262–1.588	<0.001
>84	2.026	1.813–2.265	<0.001
Marital status			
Single	1		
Married	0.652	0.586–0.727	<0.001
Widowed	0.881	0.792–0.980	0.020
Others	0.693	0.562–0.854	0.001
Level of education			
1st quartile	1		
2nd quartile	1.038	0.946–1.139	0.432
3rd quartile	1.107	1.012–1.211	0.027
4th quartile	1.218	1.108–1.338	<0.001
Unknown	1.148	0.971–1.356	0.106
Gender			
Male	1		
Female	0.935	0.877–0.996	0.038
MA in high‐risk stage II			
Chemotherapy regimen			
5‐FU	1		
Oxaliplatin	0.792	0.289–2.172	0.650
Intestinal perforation			
No	1		
Yes	2.439	1.577–3.773	<0.001
Intestinal obstruction			
No	1		
Yes	1.500	1.252–1.796	<0.001
pT category			
T3	1		
T4a	1.585	1.276–1.968	<0.001
T4b	2.610	2.055–3.314	<0.001
Age at diagnosis, years			
<70	1		
70–74	1.061	0.773–1.456	0.713
75–79	1.102	0.808–1.504	0.538
80–84	1.198	0.882–1.628	0.248
>84	2.013	1.507–2.690	<0.001
Marital status			
Single	1		
Married	0.638	0.480–0.847	0.002
Widowed	0.876	0.663–1.158	0.351
Others	0.716	0.393–1.303	0.274

MA, mucinous adenocarcinomas; NMA, nonmucinous adenocarcinomas; HCC, hierarchical condition categories; 5‐FU, 5‐fluorouracil; HR, hazard ratio; CI, confidential intervals.

An interaction analysis was performed between chemotherapy regimen (5‐FU or oxaliplatin) and histological type for patients with high‐risk stage II adenocarcinoma. No significant interaction effects were found in the test (*P *=* *0.750).

### CSS in stage III adenocarcinoma

**Figure 4:**
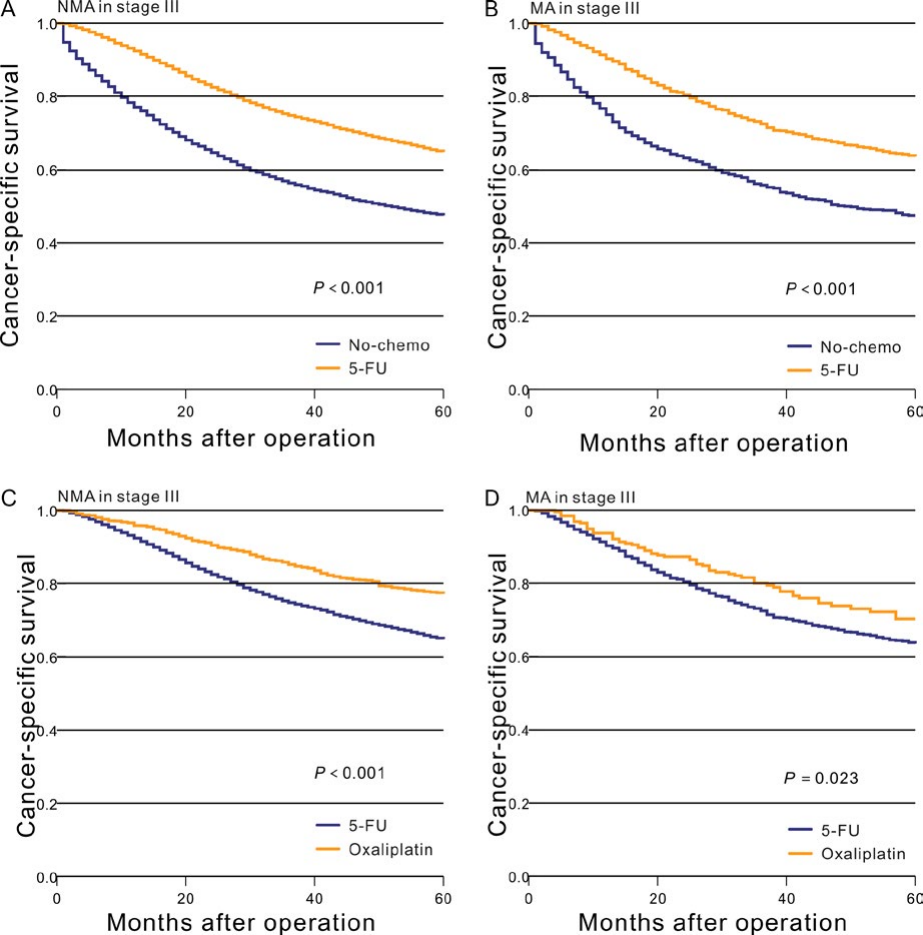
Kaplan–Meier comparison of cancer‐specific survival among patients who received different postoperative treatment stratified by histological subtype. (A) NMA in stage III (No‐chemo vs. 5‐FU); (B) MA in stage III (No‐chemo vs. 5‐FU); (C) NMA in stage III (5‐FU vs. oxaliplatin); (D) MA in stage III (5‐FU vs. oxaliplatin).

The prognosis for patients with stage III NMA in the no‐chemo group was significantly worse than the 5‐FU group (*P *<* *0.001, Fig. 4A). Similar results were also found for MA patients (*P *<* *0.001, Fig. 4B). Patients with NMA who received the oxaliplatin chemotherapy regimen had significantly improved CSS (*P *<* *0.001, Fig. 4C) compared with the 5‐FU group. Likewise, we found a survival benefit for patients with stage III MA receiving oxaliplatin compared to the 5‐FU group (*P *=* *0.023, Fig. 4D).

**Figure 5:**
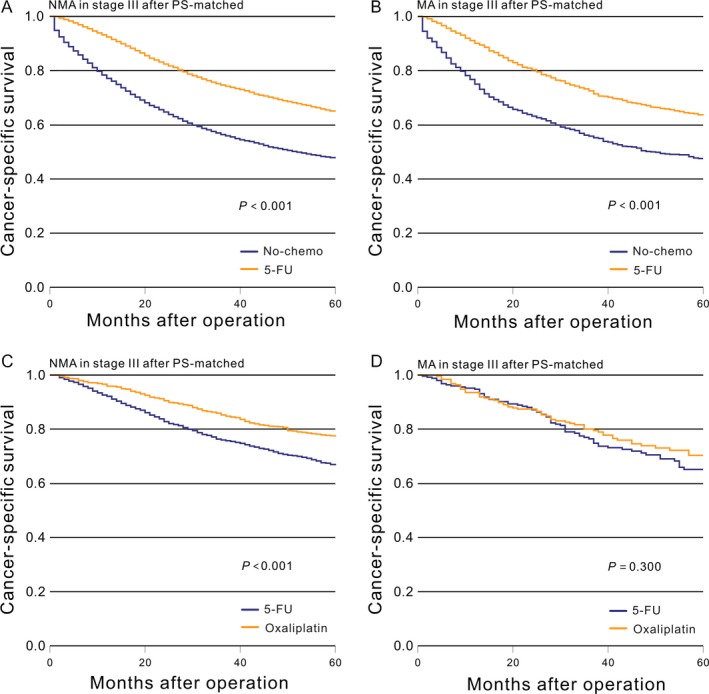
After PS‐matched, Kaplan–Meier comparison of cancer‐specific survival among patients who received different postoperative treatment stratified by histological subtype. (A) NMA in stage III (No‐chemo vs. 5‐FU); (B) MA in stage III (No‐chemo vs. 5‐FU); (C) NMA in stage III (5‐FU vs. oxaliplatin); (D) MA in stage III (5‐FU vs. oxaliplatin).

The aforementioned results were recalculated in the PS‐matched cohorts. The prognosis of patients with stage III NMA in the no‐chemo group was significantly worse than in the 5‐FU group (*P *<* *0.001, Fig. 5A). Similar results were also seen for MA patients (*P *<* *0.001, Fig. 5B). Patients with NMA who received the oxaliplatin chemotherapy regimen had significantly improved CSS (*P *<* *0.001, Fig. 5C) compared with the 5‐FU group. However, we did not find a similar survival benefit for patients with stage III MA between the oxaliplatin and 5‐FU groups (*P *=* *0.300, Fig. 5D). This result was also verified by a Cox proportional hazards model (Table 5 and 6). The result of the interaction analysis showed that there was a significant interaction effect seen in the test (*P *=* *0.040).

**Table 6:** Cox proportional hazards model for patients in stage III stratified by histological subtype

	HR	95% CI	*P*
NMA			
Chemotherapy regimen			
5‐FU	1		
Oxaliplatin	0.601	0.525–0.687	<0.001
Age at diagnosis, years			
<70	1		
70–74	0.986	0.896–1.086	0.781
75–79	1.013	0.917–1.119	0.795
80–84	1.294	1.158–1.445	<0.001
>84	1.627	1.393–1.901	<0.001
Year at diagnosis			
1992–1996	1		
1997–2000	0.905	0.822–0.997	0.044
2001–2004	0.766	0.698–0.840	<0.001
2005–2008	0.794	0.698–0.903	<0.001
HCC risk score			
1st quartile	1		
2nd quartile	1.048	0.944–1.163	0.381
3rd quartile	1.111	0.999–1.234	0.051
4th quartile	1.245	1.116–1.388	<0.001
Number of examined lymph node			
<12	1		
≥12	0.791	0.738–0.848	<0.001
pT category			
T1	1		
T2	0.965	0.718–1.298	0.815
T3	1.961	1.523–2.525	<0.001
T4a	2.691	2.062–3.510	<0.001
T4b	4.094	3.112–5.385	<0.001
Intestinal perforation			
No	1		
Yes	1.856	1.391–2.477	<0.001
Intestinal obstruction			
No	1		
Yes	1.307	1.207–1.415	<0.001
Marital status			
Single	1		
Married	0.884	0.782–1.000	0.049
Widowed	0.980	0.862–1.114	0.754
Others	0.949	0.759–1.185	0.642
Histological grade			
Well	1		
Moderate	1.160	0.975–1.381	0.094
Poor	1.461	1.221–1.749	<0.001
Undifferentiated	1.575	1.143–2.172	0.006
Unknown	1.209	0.887–1.649	0.231
Median income			
1st quartile	1		
2nd quartile	1.085	0.982–1.199	0.107
3rd quartile	1.150	1.028–1.286	0.014
4th quartile	1.155	1.006–1.326	0.041
pN category			
N1a	1		
N1b	1.360	1.244–1.486	<0.001
N2a	1.962	1.783–2.159	<0.001
N2b	3.624	3.259–4.029	<0.001
Race			
White	1		
Black	1.076	0.952–1.215	0.240
Asian	0.806	0.664–0.977	0.028
Others	0.975	0.821–1.157	0.771
Level of education			
1st quartile	1		
2nd quartile	1.041	0.939–1.154	0.445
3rd quartile	1.148	1.025–1.286	0.017
4th quartile	1.270	1.111–1.452	<0.001
Gender			
Male	1		
Female	0.970	0.897–1.049	0.445
MA			
Chemotherapy regimen			
5‐FU	1		
Oxaliplatin	0.851	0.611–1.185	0.340
Age at diagnosis, years			
<70	1		
70–74	1.136	0.890–1.450	0.306
75–79	1.243	0.975–1.586	0.079
80–84	1.602	1.226–2.094	0.001
>84	1.326	0.865–2.033	0.195
Year at diagnosis			
1992–1996	1		
1997–2000	0.867	0.685–1.098	0.236
2001–2004	0.939	0.755–1.167	0.570
2005–2008	0.707	0.510–0.981	0.038
HCC risk score			
1st quartile	1		
2nd quartile	1.114	0.876–1.417	0.378
3rd quartile	1.297	1.026–1.639	0.030
4th quartile	1.316	1.029–1.683	0.029
Number of examined lymph node			
<12	1		
≥12	0.705	0.596–0.834	<0.001
pT category			
T1	1		
T2	1.066	0.476–2.386	0.876
T3	2.038	1.048–3.965	0.036
T4a	3.166	1.589–6.308	0.001
T4b	4.793	2.358–9.742	<0.001
Intestinal obstruction			
No	1		
Yes	1.258	1.021–1.549	0.031
Marital status			
Single	1		
Married	0.846	0.624–1.148	0.284
Widowed	0.888	0.645–1.222	0.465
Others	1.561	0.951–2.560	0.078
pN category			
N1a	1		
N1b	1.286	1.025–1.614	0.030
N2a	2.176	1.718–2.756	<0.001
N2b	3.046	2.381–3.897	<0.001
Intestinal perforation			
No	1		
Yes	1.443	0.705–2.955	0.316
Histological grade			
Well	1		
Moderate	0.974	0.688–1.378	0.882
Poor	1.141	0.794–1.640	0.474
Undifferentiated	1.250	0.577–2.709	0.572
Unknown	0.864	0.555–1.345	0.517

HCC, Hierarchical condition categories; HR, hazard ratio; CI, confidential intervals; MA, mucinous adenocarcinoma; NMA, nonmucinous adenocarcinoma; 5‐FU, 5‐fluorouracil.

### FOLFOX versus CapeOx

We found no difference in survival between the FOLFOX and CapeOx group for NMA (HR: 0.817, 95% CI: 0.190–3.518, *P *=* *0.786) and MA (HR: 0.042, 95% CI: 0.001–92710.202, *P *=* *0.512) in high‐risk stage II patients. Similar results were found for NMA (HR: 1.128, 95% CI: 0.750–1.695, *P *=* *0.562) and MA (HR: 0.746, 95% CI: 0.234–2.382, *P *=* *0.618) in stage III patients. Detailed information is shown in Table 7.

**Table 7:** Univariate prognostic analysis between the FOLFOX and CapeOx groups

	HR	95% CI	*P*
NMA in low‐risk stage II	0.044	0.001–38913059	0.065
NMA in high‐risk stage II	0.817	0.190–3.518	0.786
NMA in stage III	1.128	0.750–1.695	0.562
MA in low‐risk stage II	–	–	–
MA in high‐risk stage II	0.042	0.001–92710.202	0.512
MA in stage III	0.746	0.234–2.382	0.618

MA, mucinous adenocarcinoma; NMA, nonmucinous adenocarcinoma; HR, hazard ratio; CI, confidential intervals.

## Discussion

Mucinous adenocarcinoma is a relatively common histological subtype of colon adenocarcinoma, yet the clinical significance of its histological designation remains unclear. The rate of MA was 14.1% in our study and 20–30% in previous studies 20, 21. Moreover, other studies reported that MA occurred in 10–20% cases of colon cancer 22, 23. The reason may be that the definition of MA has not been consistent across studies 24. In our study, MA was defined according to the MORPHOLOGY CODE of SEER (ICD‐O‐3: 8480). Most previous studies demonstrated worse survival in MA patients compared with NMA 25, 26. However, this is contradicted by other research 22. MA is more often discovered in the proximal colon 27, and in females 23, and it generally has a more advanced stage at presentation 27. Whether MA should be considered as an independent prognostic factor is still controversial. To the best of our knowledge, there is no difference in treatment prescribed between NMA and MA. At present, the main treatment for locally advanced colon cancer is curative resection plus chemotherapy.

Most of the benefit of postoperative chemotherapy is reported in the patients with stage III disease. The benefit of chemotherapy for stage II disease is very controversial. In our study, there was no significant difference in survival for NMA and MA patients with stage II cancer between the no‐chemo and 5‐FU groups. For patients with stage III, adjuvant chemotherapy after primary surgical treatment is usually recommended 28. As would be expected, we found a survival benefit for MA and NMA patients with stage III receiving 5‐FU compared to the no‐chemo group.

Oxaliplatin is a platinum analogue that blocks DNA replication and transcription. It has been permitted in the European Union since 1999 and in the United States since 2002 29, 30. FOLFOX had proven to be highly efficient in treatment of gastrointestinal cancer, which had enabled significant progress in clinical oncology in recent years 31. Studies have found that the 10‐year OS of patients with stage III disease receiving FOLFOX was significantly increased compared with those receiving 5‐FU alone 32. However, oxaliplatin causes severe side effects which should not be ignored. These include peripheral neuropathy and gastrointestinal side effects. The primary safety concern with oxaliplatin use is peripheral neuropathy, a cumulative dose‐related toxicity which affects 90% of all treated patients 33. Incidence of grade 3 peripheral sensory neuropathy was 12.4% for patients receiving FOLFOX and only 0.2% for patients receiving 5‐FU. Moreover, Andre et al. 34 found that neuropathy was still present in 15.4% of examined patients at 4 years post‐treatment, suggesting that oxaliplatin‐induced neuropathy may not be completely reversible in some patients.

It is quite important to identify which patients could optimally benefit from oxaliplatin treatment. This study found that patients with locally advanced colon cancer whose histological type is NMA can benefit from oxaliplatin, while those with MA cannot. We compared the prognosis of patients stratified by histological type between the 5‐FU and oxaliplatin groups. We found that in stage III and high‐risk stage II MA, adding oxaliplatin to 5‐FU in the adjuvant setting did not prolong CSS. On the contrary, the oxaliplatin regimen improved CSS in NMA patients compared with the 5‐FU regimen. The results of the PS‐match and Cox proportional hazards models in high‐risk stage II and stage III patients confirmed these findings, as do the results of the interaction analysis in stage III patients. Moreover, there were a relatively small number of patients in subgroup analyses, and therefore, more studies with a larger sample size are necessary.

In spite of this, we still could not clearly define a reason for our findings. However, we elaborated upon this phenomenon to provide some preliminary data for markers identifying the efficiency of oxaliplatin in MA, and it is important to continue researching its specific mechanism in future studies.

Our findings should be interpreted in the context of several limitations. The information on perineural, vascular, and lymphatic invasion was not available in the SEER‐Medicare database. To the best of our best knowledge, no studies to date evaluated the impact of perineural, vascular, and lymphatic invasion on the sensitivity of oxaliplatin, and no definite conclusions could be made because of limited data. Therefore, more studies are necessary to address this problem more conclusively.

The nonavailability of the microsatellite instability (MSI) status in the SEER‐Medicare database was a major limitation. It was reported that 27% of MA patients were in MSI‐H status and only 12% of NMA patients were in the MSI‐H status 35. In addition, Kim reported the prognosis of MA associated with the MSI‐H status 36. It is well known that patients with MSI‐H stage II colon cancer do not benefit from 5‐FU therapy in survival 9. In contrast, whether the MSI status can affect FOLFOX efficacy in stage III patients remains controversial. A previous study found no difference between pMMR and dMMR in survival of patients with stage III colon cancer undergoing FOLFOX adjuvant chemotherapy 37. In contrast, another study indicated that survival was significantly higher in patients undergoing FOLFOX with dMMR tumors compared to those with pMMR tumors 38. Whether the MSI status interacted with the influence of MA on the efficacy of FOLFOX needs to be better studied.

In addition, few patients were aged <65 years at the time of diagnosis in our study (3.2%), which may limit the application of these findings to younger patients with colon cancer. It was reported that the efficacy of oxaliplatin was poor for older adults 8. Therefore, we took age into account when recruiting the population for the PS‐Match analysis. Moreover, it could be also a major confounding point, in that MSI and mucinous patients seem more frequent in older population. Since that, it is important to continue researching this problem in future studies.

Finally, although both a PS‐matched technique and a Cox proportional hazards model were used to eliminate known relevant confounders, the potential for confounding based on patients selection could not be eliminated completely, as it was a retrospective exploratory study. Further prospective study was needed to verify our findings in future.

In summary, for patients with resected colon cancer who received 5‐FU‐based postoperative chemotherapy, oxaliplatin chemotherapy prolongs CSS for patients with stage III and high‐risk stage II NMA. Conversely, adding oxaliplatin to 5‐FU in postoperative chemotherapy did not improve CSS for patients with stage III or high‐risk stage II MA.

## Conflicts of Interest

The authors declare that they have no conflict of interest.

## Consent

The manuscript was approved by SEER‐Medicare for anonymity prior to submission for publication. Because the SEER‐Medicare data are de‐identified and are based on registry data, no prior informed consent was required.
